# Altered Functional Segregated Sensorimotor, Associative, and Limbic Cortical-Striatal Connections in Parkinson's Disease: An fMRI Investigation

**DOI:** 10.3389/fneur.2021.720293

**Published:** 2021-10-26

**Authors:** Tao-Mian Mi, Wei Zhang, Yu Li, Ai-Ping Liu, Zhi-Li Ren, Piu Chan

**Affiliations:** ^1^Department of Neurology, Neurobiology and Geriatrics, Beijing Institute for Brain Disorders, Xuanwu Hospital of Capital Medical University, Beijing, China; ^2^National Clinical Research Center for Geriatric Disorders, Beijing, China; ^3^Department of Neurology, The Affiliated Hospital of Xuzhou Medical University, Xuzhou, China; ^4^Department of Electronic Science and Technology, University of Science and Technology of China, Hefei, China; ^5^Clinical Center for Parkinson's Disease, Capital Medical University, Beijing, China; ^6^Key Laboratory for Neurodegenerative Disease of the Ministry of Education, Beijing Key Laboratory for Parkinson's Disease, Beijing, China; ^7^Advanced Innovation Center for Human Brain Protection, Capital Medical University, Beijing, China

**Keywords:** Parkinson's disease, habitual control, goal-directed behavior, cortical-striatal loop, functional connectivity

## Abstract

Multiple studies have identified segregated functional territories in the basal ganglia for the control of goal-directed and habitual actions. It has been suggested that in PD, preferential loss of dopamine in the posterior putamen may cause a major deficit in habitual control (mediated by the sensorimotor cortical-striatal loop), and the patients may therefore be forced into a progressive reliance on the goal-directed behavior (regulated by the associative cortical-striatal loop). Functional evidence supporting this point is scarce at present. This study aims to verify the functional connectivity changes within the sensorimotor, associative, and limbic cortical-striatal loops in PD. Resting-state fMRI of 70 PD patients and 30 controls were collected. Bilateral tripartite functional territories of basal ganglia and their associated cortical structures were chosen as regions of interest, including ventral striatum and ventromedial prefrontal cortex for limbic loop; dorsomedial striatum and dorsolateral prefrontal cortex for associative loop; dorsolateral striatum and sensorimotor cortex for sensorimotor loop. Pearson's correlation coefficients for each seed pair were calculated to obtain the functional connectivity. The relationships between functional connectivity and disease severity were further investigated. Functional connectivity between dorsolateral striatum and sensorimotor cortex is decreased in PD patients, and negatively correlated with disease duration; whereas functional connectivity between dorsomedial striatum and dorsolateral prefrontal cortex is also decreased but postitively correlated with disease duration. The functional connectivity within the sensorimotor loop is pathologically decreased in PD, while the altered connectivity within the associative loop may indicate a failed attempt to compensate for the loss of connectivity within the sensorimotor loop.

## Introduction

Parkinson's disease (PD) is the second most common neurodegenerative disease (1). Since the original “direct vs. indirect pathway” model of basal ganglia was introduced, it has spawned many valuable experimental studies and clinical developments in PD (2, 3). With a lot of new anatomical, functional, and clinical evidence, however, it is increasingly perceived as having limitations (4). Multiple human and animal studies have identified spatially segregated functional territories in the basal ganglia for the control of goal-directed and habitual actions (4–7). Specifically, the basal ganglia is regionally segregated into a tripartite functional territories, wherein the dorsolateral striatum (posterior putamen) is engaged in sensorimotor functions, the dorsomedial striatum (caudate and anterior putamen) in associative functions, and the ventral striatum (nucleus accumbens and olfactory tubercle) in limbic functions. The consequent division of the basal ganglia into a limbic, associative, and sensorimotor networks has provided a functional framework, onto which different kinds of associative processing have been successfully mapped (7, 8). The limbic loop connecting the ventral striatum with the ventromedial prefrontal cortex (vmPFC) has been implicated in motivational and emotional processing; whereas the associative and sensorimotor networks regulate different forms of behaviors in instrumental behavior processing. Respectively, the associative network connecting the dorsomedial striatum with dorsolateral prefrontal cortex (dlPFC) mainly contributes to the goal-directed behaviors (7, 9), while the sensorimotor network projecting from the dorsolateral striatum to the sensorimotor cortex is mainly responsible for the habitual control behaviors in instrumental learning (7, 9).

Progressive loss of the ascending dopaminergic projection in the basal ganglia is one of the fundamental pathological features in PD (1), predominantly in the posterior putamen, namely the sensorimotor territory associated with the habitual control behaviors. Based on this information, researchers have thus proposed that the preferential loss of dopamine in the sensorimotor territory of the basal ganglia may cause a major deficit in habitual control in PD patients, and the patients may therefore be forced into a progressive reliance on the goal-directed behavior that is mediated by comparatively preserved processing in the dorsomedial striatum (4). However, this theory has never been proved in human studies. The aim of the present study is to verify such theory indirectly by investigating the functional connectivity changes between the functionally segregated territories of basal ganglia and their associated functional cerebral cortex among the limbic, associative, and sensorimotor loops in PD patients.

Till now, multiple fMRI studies have investigated resting-state functional connectivity within the basal ganglia in PD patients. Helmich et al. (10) demonstrated that PD patients have decreased coupling between the posterior putamen and the inferior parietal cortex, while the latter region show increased functional connectivity with the anterior putamen. Several other studies found reduced correlations between sensorimotor cortex and visual regions (11), and posterior putamen (12) in PD patients. Besides, the striatal functional connectivity with the temporal, parietal, occipital and cerebellar regions was found to be increased in both drug-naïve and under optimized dopaminergic treatment PD patients (13). It is worth noting that, almost all the aforementioned studies are based on data-driven analyses, which reveal heterogeneous patterns of functional connectivity alterations. A hypothesis-driven approach that specifically investigates the functional connectivity within the tripartite functional circuits corresponding to goal-directed and habitual control, however, has not been investigated yet. In the present study, utilizing resting-state fMRI techniques, we applied hypothesis-driven analyses to specifically look at the functional connectivity changes among the limbic, associative, and sensorimotor loops in PD patients. We hypothesized that, as suggested previously, the functional connections within the sensorimotor loop would be pathologically decreased, whereas the connections within the associative loop would be increased as a result of the compensatory effect. We note that, in the study performed by Helmich et al. (10), the authors segmented the basal ganglia into the posterior putamen, the anterior putamen, and caudate as regions of interest (ROIs), and then performed the connectivity patterns with the rest of the anatomical brain regions from the automated anatomical labeling (AAL) atlas, which is different from our approach used in the present study. Here, to verify our hypothesis, we defined the tripartite functional territories of basal ganglia (ventral striatum consisting of nucleus accumbens and olfactory tubercle, dorsomedial striatum consisting of caudate and anterior putamen, and dorsolateral striatum consisting of posterior putamen) and their related functional cortical cortex (vmPFC, dlPFC and sensorimotor cortex) as our ROIs (see more details in Methods).

## Materials and Methods

### Participants

Seventy patients diagnosed with idiopathic PD according to the UK Brain Bank Clinical Criteria were recruited from the Movement Disorders Clinic of the Xuanwu Hospital between August 2017 and December 2019. Exclusion criteria were: (i) presence of contraindications for resting-state fMRI; (ii) history of deep brain stimulation surgery; (iii) marked rest tremor; (iv) comorbidities of neurological disease other than PD; (v) left-handedness. In addition, a group of 30 sex- and age-matched healthy volunteers were recruited as healthy controls (HC). The demographic and clinical characteristics of all participants are shown in Table 1. The experiments were performed according to the Declaration of Helsinki and were approved by the Institutional Review Board of Xuanwu Hospital of Capital Medical University. Written informed consent was obtained from all subjects prior to the study.

**Table 1:** Demographics and clinical features of participants.

**Features**	**PD group (*N* = 70)**	**HC group (*N* =30)**	***T***	***P***
Gender (m/f)	41/29	16/14	0.235	0.628
Age	60.61 ± 9.68 (44–80)	58.30 ± 7.45 (43–73)	1.168	0.246
H–Y stage	2.29 ± 0.79 (1.0–4.0)	–	–	–
MDS–UPDRS III	37.40 ± 17.74 (10–72)	–	–	–
Disease duration	6.54 ± 4.49 (1–15)	–	–	–
LEDD	586.61 ± 396.74 (175–1698)	–	–	–
MMSE	28.13 ± 2.01 (22–30)	28.57 ± 1.45 (25–30)	1.076	0.285
MoCA	25.21 ± 3.75 (18–30)	26.13 ± 3.52 (17–30)	1.144	0.255
**Head motion**				
Translation X (mm)	0.31 ± 0.23 (0.04–1.04)	0.36 ± 0.35 (0.03–1.63)	0.881	0.381
Translation Y (mm)	0.25 ± 0.17 (0.05–0.86)	0.24 ± 0.09 (0.05–0.45)	0.196	0.899
Translation Z (mm)	0.35 ± 0.36 (0.06–2.50)	0.33 ± 0.30 (0.05–1.45)	0.203	0.840
Rotation X (degrees)	0.78 ± 0.75 (0.14–2.65)	0.62 ± 0.52 (0.11–2.52)	1.008	0.316
Rotation Y (degrees)	0.63 ± 0.51 (0.05–2.53)	0.52 ± 0.39 (0.07–1.54)	0.925	0.356
Rotation Z (degrees)	0.63 ± 0.50 (0.09–2.09)	0.48 ± 0.40 (0.07–1.98)	1.293	0.199

*Means and SD are shown for continuous variables. H–Y stage, Hoehn and Yahr stage; MDS–UPDRS III, Movement Disorder Society–Unified Parkinson's Disease Rating Scale motor score; LEDD, levodopa equivalent daily dose; MMSE, Mini–mental State Examination; MoCA, Montreal Cognitive Assessment*.

### Clinical Assessments

For PD patients, clinical assessments were conducted in their practical “OFF” state, that is, at least after a 12-h withdrawal of anti-parkinson medication. The Movement Disorder Society Unified Parkinson's Disease Rating Scale motor scores (MDS-UPDRS III) and Hoehn and Yahr (H-Y) stage were used to assess motor disability and disease severity. The Mini-mental State Examination (MMSE) and Montreal Cognitive Assessment (MoCA) (14) scales were adopted to assess general cognitive function for all participants.

### Resting-State fMRI Data Acquisition

Imaging was carried out in a SIEMENS Trio 3T scanner. During scanning, participants were asked to keep their head still and eyes closed, but not fall asleep. Earplugs and a head coil with foam pads were used to minimize machine noise and head motion. For PD patients, fMRI scans were acquired at OFF state, following a 12-h period of medication withdrawal. We also acquired high-resolution T1 weighted anatomical images for each participant, and a radiologist assessed the images to exclude participants with other neurological pathology. Structural images were acquired using a sagittal magnetization prepared rapid gradient echo three-dimensional T1-weighted sequence (repetition time [TR] = 1,970 ms, echo time [TE] = 3.9 ms, inversion time [TI] = 1,100 ms, flip angle [FA] = 15°). Blood-oxygen-level-dependent images were obtained using the following SE-EPI sequence: repetition time = 2,000 ms, echo time = 30 ms, slice thickness/gap = 4.0/0 mm, axial slices = 33 layers, flip angle = 90°, FOV = 256 ×256 mm, matrix size = 64 ×64, and scanning time = 8 min.

### Data Preprocessing

Data were preprocessed and analyzed using DPABI version 2.2 (http://rfmri.org/dpabi) and SPM12 software package (http://www.fil.ion.ucl.ac.uk/spm), as used in our previous study (15). Briefly, the first 10 time points were discarded to allow participants to get used to the scanning noise and the magnetization to approach a dynamic equilibrium. The remaining images were corrected for slice timing and realigned to remove excessive head motion. EPI data were then normalized into a standard brain space template, and resampled to 3.0 ×3.0 ×3.0 mm isotropic voxels. Linear trends were removed and images were smoothed with a 6-mm Gaussian kernel to increase the signal-to-noise ratio. Friston-24 correction (16) was further adopted to reduce potential confounds of head motion. Then the nuisance covariates of cerebrospinal fluid signal and white matter signal were regressed-out to reduce possible effects of physiological artifacts. Participants were excluded from analysis if their head motion (mean frame-wise displacement Jenkinson) was greater than mean+2^*^SD (threshold 0.30 mm) (17), which led to an exclusion of four participants, including three PD patients and one healthy subject.

### Regions of Interest

**Figure 1:**
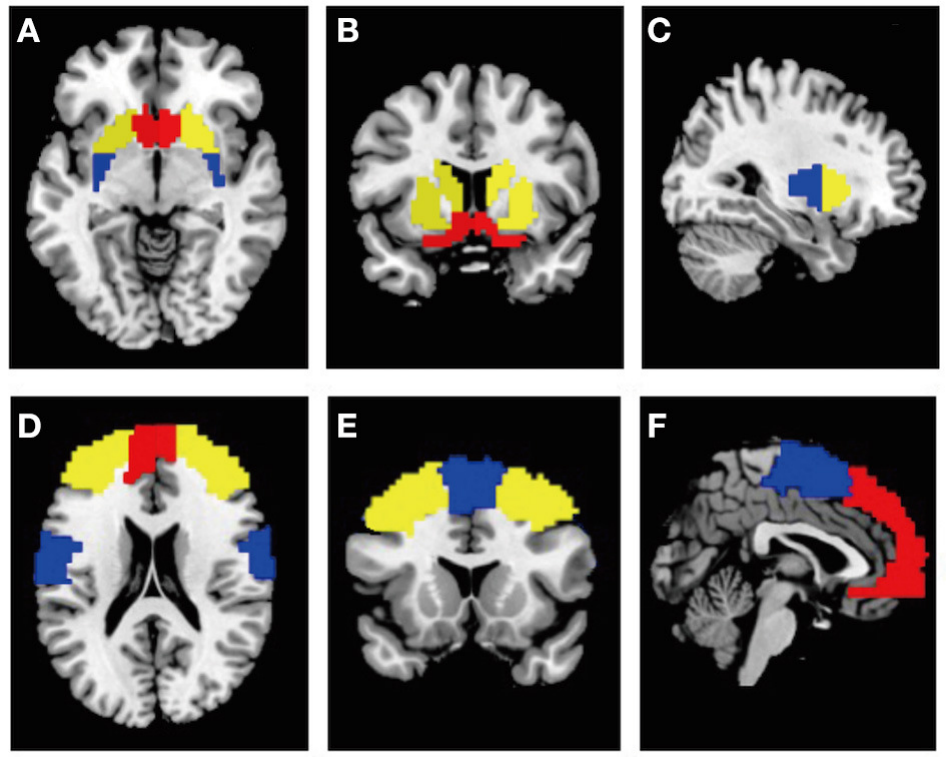
**(A–C)** ROIs of the bilateral tripartite functional territories of striatum from the horizontal, coronal, sagittal plane view. Red: ventral striatum; yellow: dorsomedial striatum; blue: dorsolateral striatum. **(D–F)** ROIs of their associated cortical structures from the horizontal, coronal, sagittal plane view. Red, vmPFC; yellow, dlPFC; blue, sensorimotor cortex.

In the present study, twelve ROIs were chosen for the functional connectivity analysis, including the bilateral tripartite functional territories of basal ganglia and their associated cortical structures: ventral striatum (Figures 1A,C in red: consists of nucleus accumbens and olfactory tubercle) and vmPFC (Figures 1D–F in red) for limbic processing; dorsomedial striatum (Figures 1A–C in yellow: consists of caudate and anterior putamen) and dlPFC (Figures 1D–F in yellow) for goal-directed behavior; dorsolateral striatum (Figures 1A,C in blue: posterior putamen) and sensorimotor cortex (Figures 1D–F in blue) for habitual-control. The masks for all ROIs were derived from the automatic anatomical labeling atlas 3 (18). Anterior vs. posterior putamen was demarcated at MNI-coordinate y = 2 (anterior>2; posterior <2) (19). The mask for the sensorimotor cortex was consisted of “Supplementary motor area,” “Postcentral gyrus,” and “Precentral gyrus,” vmPFC covering the medial prefrontal cortex were obtained from “Superior frontal gyrus, medial” and “Superior frontal gyrus, medial orbital” (19), and dlPFC covering the dorsolateral prefrontal cortex were derived from “Superior frontal gyrus, dorsolateral” and “Middle frontal gyrus” (20). The mean time course of each ROI was extracted to represent ROI activity.

### Functional Connectivity Computation

For each participant, functional connectivity between each pair of ROIs were estimated by calculating the Pearson's correlation coefficients between their mean time courses, resulting in a 12 ×12 functional connectivity matrix. To further improve the normality of correlation distribution, individual correlation coefficients were subsequently transformed to *z*-values using the Fisher *r*-to-*z* transformation.


$$\begin{array}{c} FC\left(i,j\right)=\frac{cov\left(i,j\right)}{std\left(i\right)*std\left(j\right)}\\ FC{\left(i,j\right)}_{z}=0.5*log\left(\frac{1+FC\left(i,j\right)}{1-FC\left(i,j\right)}\right) \end{array}$$

Where and represent the functional connectivity and Fisher's z-transformed functional connectivity between ROI and ROI, respectively, and are, respectively, the standard deviations of mean time courses in ROI and ROI, and is the covariance of these mean time courses.

### Statistical Analysis

Demographic data were presented as mean ± SD for continuous variables. Independent two-sided *t*-test was performed for the comparison of continuous variables, and the χ^2^-test was used to compare categorical variables. The normality was tested using Shapiro-Wilk's test. The threshold for the level of significance was set at α = 0.05. All statistical analyses were performed using Stata 15.1.

For the functional connectivity analyses, after examining the normality of distribution for all functional connectivity pairs, we then compared the strengths of functional connectivity between the PD and HC group using a two-sample *t*-test, with age, sex and head motion parameters included as covariates. Furthermore, we performed Pearson's correlation analysis to explore the relationship between functional connectivity strength and disease severity (MDS-UPDRS III scores and disease duration). False discovery rate correction was applied in the current study for multiple comparison correction.

## Results

### Clinical and Demographic Characteristics

Participant demographics and clinical features are shown in Table 1. For PD patients, the mean MDS-UPDRS III scores were 37.40 ± 17.74 points, with a mean H-Y stage of 2.29 ± 0.79 and a mean disease duration of 6.54 ± 4.49 years. There were no significant differences in gender, age, MMSE, and MoCA scores between the two groups. In addition, no significant differences were found in head motion parameters between the PD patients and healthy controls (0.117 ± 0.084 mm vs. 0.106 ± 0.009 mm, *p* = 0.507).

### Functional Connectivity Strength

**Figure 2:**
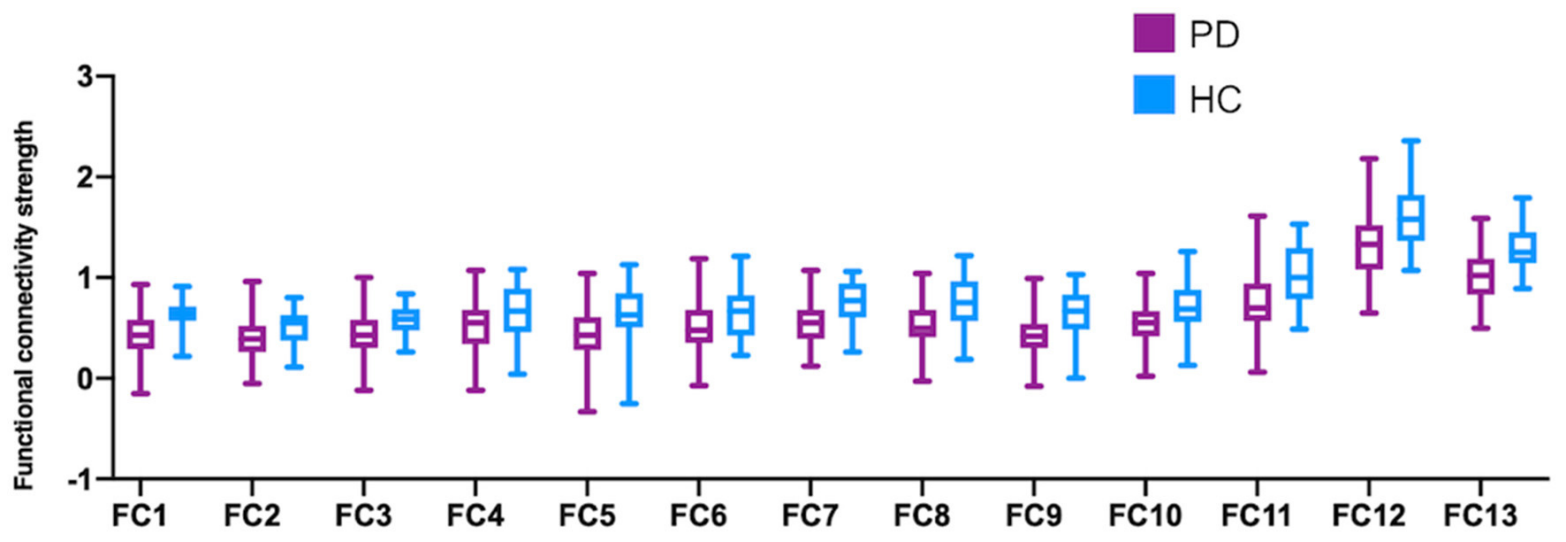
Functional connectivity strength comparisons between PD and HC.

Among all the 66 (12^*^11/6 = 66) pairs of possible connections, thirteen were detected to be significant after FDR correction in the comparison between PD patients and healthy controls (Figure 2). As demonstrated in Table 2, compared to healthy subjects, PD patients show significantly decreased functional connectivity within the sensorimotor loop (between the left dorsolateral striatum and bilateral sensorimotor cortex, as well as between the right dorsolateral striatum and left sensorimotor cortex). Contrary to what we expected, however, the functional connectivity within the associative loop (between the left dorsomedial striatum and bilateral dlPFC, as well as right dorsomedial striatum and left dlPFC) was also significantly decreased in PD patients. In addition, PD patients have significantly weaker functional communication between the bilateral dorsomedial striatum and bilateral dorsomedial striatum. Significantly decreased functional connectivity within the bilateral dorsolateral striatum pair, dorsomedial striatum pair, and sensorimotor cortex pair was also found in the PD group. No significant group difference was observed with regards to the functional connectivity involving the ventral striatum.

**Table 2:** Functional connectivity strength comparisons between PD and HC.

	**Seed pairings**	**PD**	**HC**	***T***	***P***
FC1	Dorsomedial striatum_L-dlPFC_L	0.43 ± 0.23	0.62 ± 0.16	−3.945	0.000^*^
FC2	Dorsomedial striatum_L-dlPFC_R	0.40 ± 0.20	0.51 ± 0.17	−2.765	0.007^*^
FC3	Dorsomedial striatum_R-dlPFC_L	0.44 ± 0.22	0.58 ± 0.16	−3.236	0.002^*^
FC4	Dorsolateral striatum_L-Sensorimotor_cortex_L	0.52 ± 0.26	0.67 ± 0.27	−2.963	0.004^*^
FC5	Dorsolateral striatum_L-Sensorimotor_cortex_R	0.45 ± 0.26	0.63 ± 0.28	−3.374	0.001^*^
FC6	Dorsolateral striatum_R-Sensorimotor_cortex_L'	0.50 ± 0.26	0.65 ± 0.26	−2.752	0.007^*^
FC7	Dorsolateral striatum_L-Dorsomedial striatum_L	0.53 ± 0.20	0.75 ± 0.22	−4.537	0.000^*^
FC8	Dorsolateral striatum_L-Dorsomedial striatum_R	0.53 ± 0.21	0.75 ± 0.25	−4.192	0.000^*^
FC9	Dorsolateral striatum_R-Dorsomedial striatum_L	0.42 ± 0.18	0.63 ± 0.26	−4.333	0.000^*^
FC10	Dorsolateral striatum_R-Dorsomedial striatum_R	0.55 ± 0.19	0.69 ± 0.26	−2.671	0.009^*^
FC11	Dorsolateral striatum_R-Dorsolateral striatum_L	0.76 ± 0.28	1.01 ± 0.29	−4.035	0.000^*^
FC12	Sensorimotor_cortex_R-Sensorimotor_cortex_L	1.33 ± 0.31	1.58 ± 0.29	−3.506	0.001^*^
FC13	Dorsomedial striatum_R-Dorsomedial striatum_L	1.02 ± 0.23	1.29 ± 0.23	−5.283	0.000^*^

*Means and SD are shown for the functional connectivity strength (z-scores). dlPFC, dorsolateral prefrontal cortex.*

* *p < 0.01 after FDR correction*.

### Relationships Between Functional Connectivity and Clinical Severity

**Figure 3:**
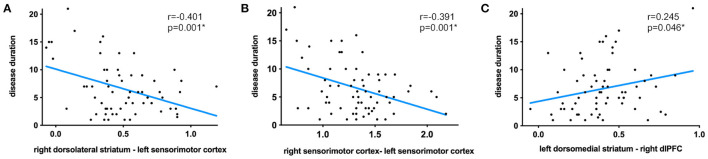
Significant correlations between disease duration and functional connectivity strength between the right dorsolateral striatum–left sensorimotor cortex pair **(A)**, bilateral sensorimotor cortex pair **(B)**, and left dorsomedial striatum-right dlPFC pair **(C)**.

Seed pairings demonstrating significant group differences in functional connectivity strength were further analyzed for associations with clinical severity, including the MDS-UPDRS III scores and disease duration (see Table 2 for all significant relationships). Results showed that functional connectivity between the right dorsolateral striatum–left sensorimotor cortex pair (Figure 3A: *r* = −0.401; *p* = 0.001), as well as between the bilateral sensorimotor cortex pair (Figure 3B: *r* = −0.391; *p* = 0.001) was negatively correlated with disease duration. Conversely, longer disease duration was strongly correlated with higher functional connectivity between the left dorsomedial striatum-right dlPFC pair (Figure 3C: *r* = 0.245; *p* = 0.046). The MDS-UPDRS III scores were not related to functional connectivity strength for any of the identified seed pairings. Moreover, we further performed the correlations with MoCA scores, but did not find any significant correlation.

## Discussion

An impressive array of studies have demonstrated that medial frontal cortex and dorsal striatum are anatomically connected (21, 22) and identified distinct, spatially-segregated regions in the basal ganglia for the control of goal-directed and habitual actions (4, 7). Studies in rodents indicated that the region of dorsomedial striatum and medial prefrontal were involved in the goal-directed actions (23, 24), whereas the region of dorsolateral striatum and premotor cortex were responsible for habitual control behavior (5, 6). Miyachi and colleagues (25, 26) provided evidence for a homologous functional organization within the non-human primate, whereby the caudate and rostral putamen correspond to the rodent dorsomedial striatum, and the caudal putamen to the rodent dorsolateral regions, respectively. In the current study, we investigated the pattern of functional connectivity between the functionally segregated territories of basal ganglia and their associated functional cerebral cortex among the limbic, associative, and sensorimotor loops in PD patients, that are suggested to be associated with goal-directed and habitual control in PD patients. Consistent with prior knowledge, our results demonstrated that functional connections within the sensorimotor loop (dorsolateral striatum and sensorimotor cortex pair) are pathologically decreased in PD patients; whereas contrary to our hypothesis, the connections within the associative loop (dorsomedial striatum and dlPFC pair) was also decreased. However, correlation results showed that longer disease duration is associated with stronger connections within the associative loop. These findings indicate that the observed pattern of altered connectivity within the associative loop may be reflective of a failed attempt to compensate for the loss of connectivity within the sensorimotor loop.

During the last decade, resting-state fMRI studies have been extensively applied in understanding the pathophysiology for multiple neurodegenerative disorders, including PD. This non-invasive method infers neural activity from spontaneous blood-oxygen-level-dependent (BOLD) signal fluctuations (27). Functional connectivity can be inferred from spatially distinct brain regions during resting state, indicating the possible intrinsic correlated patterns (28, 29). In PD patients, functional connectivity changes involving the cortico-striatal pathway have been consistently reported, with a clinical correlation with disease severity, suggesting that functional disruption within this network and all its associated hubs may be related with the neuropathological process underlying PD (30). In the present study, we mainly focused on exploring the pattern of functional connectivity changes within the limbic, associative and sensorimotor loops in PD patients. Consistent with previous studies, our results also demonstrate diffuse disrupted and weakened functional connections within the cortico-striatal network, mainly within the associative and sensorimotor loops, which are responsible for the goal-directed and habitual control behaviors in instrumental learning (7, 9).

Researchers have suggested that preferential loss of dopamine in the sensorimotor territories of PD patients may cause a major deficit in habitual control, and pathological output from such dysfunctional sensorimotor circuitry will distort the expression of residual goal-directed responses (4). Direct evidence supporting this theory is, however, scarce at present. To expand our knowledge on it, we investigated the brain connectivity between functional segregated sub-regions of basal ganglia and their associated cortical structures which may better describe the alterations of cortical-striatal connections. Consistent with our hypothesis, we found that the functional connectivity within the sensorimotor loop is significantly weakened in PD patients, which is also negatively correlated with disease duration, indicating a pathological effect of PD. With dopaminergic degeneration developing gradually, pathological impairments are more severe as disease progresses (1). Indeed, PD patients' ability to carry on performing normal habitual components of actions (for example, blinking, arm-swinging, facial expressions, pacing of gait), which is mediated by the sensorimotor loop, is impaired and this ability is further aggravated with disease progression (31). Despite prior speculation that the functional connections within the associative loop would increase as a result of the compensatory effect, our results demonstrated that the functional connectivity within the associative loop was decreased in PD patients. Nevertheless, the functional connectivity within the associative loop was shown to be positively correlated with disease duration, which may indicate an attempt to compensate for the loss of functional connectivity within the sensorimotor loop. It is suggested that with a progressive loss habitual processing, PD patients would rely increasingly on the goal-directed control system which is mediated by the associative loop (4). For instance, PD patients require a conscious decision to start walking and they stop abruptly if they are distracted by a different external stimulus, a new idea or another behavior (e.g., talking) (32). However, de Wit and colleagues found a disease severity–dependent deficit in goal-directed behavior in PD patients (33). It is likely that the compensatory effect strengthens at a relatively early stage, presumably, but may diminish or eventually fail as pathological damages become more severe with disease progression (34).

In addition to the decreased functional connectivity within the associative and sensorimotor loops, compared to healthy subjects, PD patients also exhibited significantly weaker functional connectivity in the bilateral dorsolateral striatum, dorsomedial striatum, and sensorimotor cortex pair, indicating impaired functional coordination between homotopic brain regions in PD patients. Moreover, the functional connectivity between the bilateral sensorimotor cortex pair was negatively correlated with disease duration. These findings are consistent with a previous study showing that PD patients exhibited significantly lower voxel-mirrored homotopic connectivity in the putamen and sensorimotor cortical regions (35).

Most of the movement disorders of PD can be attributed to impaired automatic habitual performance (4, 36), which would revert to goal-directed system as a compensatory recruitment. For example, with an attempt to explain freezing of gait (FOG) from a cognitive-based perspective, Vandenbossche et al. conducted a study focusing on dysfunction in automaticity and control, mediated by the frontostriatal circuits (37). In their study, PD patients without FOG may compensate for disorders in automaticity by means of increasing cognitive control which can't be seen in PD patients with FOG, suggesting cognitive compensation would be broken down when both automatic and controlled processes were more severely impaired. In light from above, perhaps we can to some extent explain why the functional connectivity within the associative loop was shown to be decreased in PD patients.

In conclusion, in this study, we investigated the brain connectivity alterations in resting state that are suggested to be associated with goal-directed and habitual control in PD patients. Our findings demonstrate that the functional connectivity within the sensorimotor loop is pathologically decreased in PD patients, while the observed pattern of altered connectivity within the associative loop may indicate a failed attempt to compensate for the loss of connectivity within the sensorimotor loop. We note that, however, the present study is a preliminary investigation of the goal-directed vs. habitual control in PD patients utilizing the resting-state fMRI technique, providing additionally insights from brain connectivity perspective. Further task-based fMRI studies involving specific paradigms clarifying the goal-directed and habitual control behaviors in instrumental learning are warranted to illuminate their underlying mechanisms.

## Data Availability Statement

The raw data supporting the conclusions of this article will be made available by the authors, without undue reservation.

## Ethics Statement

The studies involving human participants were reviewed and approved by the Institutional Review Board of Xuanwu Hospital of Capital Medical University. The patients/participants provided their written informed consent to participate in this study.

## Author Contributions

TMM and PC designed the study. TMM and WZ carried out data collection. TMM, WZ, YL, and APL analyzed the data. TMM and WZ drafted the manuscript. PC and APL revised the manuscript. All authors read and approved the final version for publication.

## Funding

This work was supported by the grants from the National Key R&D Program of China (Nos. 2018YFC1312001, 2017YFC0840105, and 2017ZX09304018), Key Realm R&D Program of Guangdong Province (2018B030337001), and National Natural Science Foundation of China (81903822 and 61701158).

## Conflict of Interest

The authors declare that the research was conducted in the absence of any commercial or financial relationships that could be construed as a potential conflict of interest.

## Publisher's Note

All claims expressed in this article are solely those of the authors and do not necessarily represent those of their affiliated organizations, or those of the publisher, the editors and the reviewers. Any product that may be evaluated in this article, or claim that may be made by its manufacturer, is not guaranteed or endorsed by the publisher.
